# External Application of Croton Oil

**Published:** 1842-12

**Authors:** 


*External Application of Croton Oil.*—Whenever it is required to use this method of counter-irritation, M. Bouchardat strongly recommends a plaster which has been much used by M. Chomel at the Hôtel Dieu, and which is thus prepared: Four parts of diachylon-plaster are melted at a very gentle heat, and while it is half liquid one part of croton oil is mixed with it, and the mixture is then spread in a thick layer on calico. Pieces cut from this may be applied to the skin, like ordinary sticking-plaster, and quickly produce an active irritation.—*B. & F. Med. Rev.* July 1842, from *Bull. Gén. de Thérapeutique*, March 1842.

